# Impact of Stress on the Prevalence of Acne Among Medical Students in the Middle East: A Systematic Review

**DOI:** 10.7759/cureus.90815

**Published:** 2025-08-23

**Authors:** Lamiss A Alzahrani, Nuha A Alfurayh, Norah S Jadaan, Rana S Alqahtani, Batul A Alqahtani

**Affiliations:** 1 General Practice, Riyadh Second Health Cluster, Riyadh, SAU; 2 Dermatology, Imam Abdulrahman Alfaisal Hospital, Riyadh, SAU; 3 College of Medicine, King Khalid University, Abha, SAU

**Keywords:** acne vulgaris, medical school students, middle east, prisma 2020, systematic literature review, academic stress

## Abstract

Acne vulgaris is a prevalent dermatological condition among young adults, with stress frequently implicated as a contributing factor. Medical students, due to their academic demands, are particularly susceptible to both stress and acne. This systematic review aims to evaluate the impact of stress on the prevalence and severity of acne among medical students in the Middle East. This review followed the Preferred Reporting Items for Systematic Reviews and Meta-Analyses guidelines. A comprehensive literature search was conducted in PubMed, Scopus, Web of Science, CINAHL Ultimate, and Google Scholar. Eligible studies included those that examined the association between stress and acne among medical students. In total, 11 studies met the inclusion criteria, encompassing 3,063 participants, primarily medical students with an average age of 21 years. Most studies (n = 9) were cross-sectional, with one case-control and one prospective study. The pooled prevalence of acne was 64.3%. Nine studies reported a statistically significant association between stress and acne severity (p < 0.05), while the remaining studies noted a non-significant but suggestive relationship. Common assessment tools included the Perceived Stress Scale, Global Acne Grading System, and Dermatology Life Quality Index. The findings support a potential bidirectional relationship between psychological stress and acne flare-ups. There is consistent evidence suggesting that stress significantly contributes to the prevalence and severity of acne among medical students in the Middle East. Early stress management interventions may help mitigate acne severity in this population.

## Introduction and background

Acne vulgaris is a widespread inflammatory skin disorder. It affects individuals of all ages and ethnic backgrounds and arises from inflammation of the pilosebaceous unit, which comprises a hair follicle and its associated sebaceous gland. Several factors contribute to this inflammation. These factors include excessive sebum production, bacterial colonization, hormonal fluctuations, and genetic predispositions [1]. Clinically, acne presents with a variety of lesions such as comedones, papules, pustules, nodules, and, in severe cases, scarring. These lesions commonly affect the face, neck, chest, and back. They also vary in distribution and severity among individuals [2]. Early intervention and adherence to treatment regimens are crucial to prevent complications of this condition. Beyond its physical manifestations, acne significantly impacts patients’ psychological well-being, as many individuals experience diminished self-esteem, social withdrawal, and symptoms of depression, largely due to the visible nature of the condition [1].

According to the Global Burden of Disease study, acne is the eighth most common skin disease and affects 9.38% of all age groups [3]. Acne prevalence is notably high among medical students in the Middle East and other regions. It ranges from approximately 34% to nearly 98% in various studies [4]. For instance, according to a study by Alrabiah et al., which included medical sciences students, reported that 78.5% of the students suffered from acne [5]. Similarly, studies from Syria and Malaysia reported acne prevalence rates of 34.7% and 75.8%, respectively, with female students often experiencing more severe forms of acne [6,7]. The interaction between skin and the nervous system is complicated. It has long been assumed that stress can cause or worsen acne [8]. Stress is the body’s natural reaction to any pleasant or unpleasant demand. Medical students endure extra psychological stress from academic demands, sleep deprivation, clinical responsibilities, or social expectations [9].

This can create a complex bidirectional relationship between dermatological and mental health challenges. Psychological stress activates the hypothalamic-pituitary-adrenal axis and causes some biological consequences. Due to the release of corticotropin-releasing hormone receptors, the skin forms acne in response to stressful situations [10]. A multicenter study involving 17 Korean hospitals reported that 82% of the participants had acne due to psychological stress [11]. Previously, a study by Almojali et al. from Saudi Arabia highlighted the significant stress that medical students endure and linked it with sleep disturbances [9]. Furthermore, a study at the University of Melbourne in Australia, which included final-year medical students, discovered that 67% of the students identified stress as an aggravating factor for acne [8]. Previous studies have reported a correlation between an increase in the intensity of stress and an increase in the severity of acne among the female medical students of Saudi Arabia [12].

Additional factors also compound the impact of stress on acne among medical students. These factors can be dietary habits rich in high glycemic index foods and fast food consumption, environmental factors such as heat and humidity, and lifestyle behaviors such as sleep deprivation [8]. The interplay of these factors with stress creates a complex environment that promotes acne development and persistence [5,13]. Despite the insights provided in the literature regarding the impact of stress on the development of acne, contradictions persist that underscore the need for more standardized studies. Furthermore, there is a paucity of summarized research on acne and stress among medical students in the Middle East. This leaves a gap in the literature. This systematic review aims to comprehensively analyze the current evidence on the impact of stress on acne prevalence among medical students in Middle Eastern countries.

## Review

Methodology

This systematic review was conducted according to the guidelines prescribed by the Preferred Reporting Items for Systematic Reviews and Meta-Analyses (PRISMA) [14].

Search Strategy

A systematic search was conducted in PubMed, CINAHL Ultimate, Scopus, and Web of Science. Apart from these databases, Google Scholar was also searched for relevant studies. The search was completed by using various keywords such as “stress,” “acne,” “acne vulgaris,” and “Middle East.” These keywords were combined by Boolean operators OR and AND. The detail of the keywords is presented in the Appendices.

Study Eligibility

The PICOS for the systematic review included population (P): studies involving medical students; interventions (I): stress; comparators (C): no stress group; outcomes (O): prevalence of acne, association with stress, and study design; (S): randomized controlled trials (RCTs), cohort studies, case-control studies, and cross-sectional studies. The exclusion criteria included studies not reporting outcomes related to acne and stress among medical students. Furthermore, non-human studies, case reports, reviews, and opinion articles were also excluded. Non-English-language studies without available translations were also excluded.

Study Selection

The search results from databases were transferred to the reference manager (EndNote 20, Thomson Reuters). The results were combined and uploaded to Rayyan, a web-based software [15]. Before screening, duplicates were removed. During the screening process, four independent reviewers were involved. The reviewers were divided into two groups, with group A involving two reviewers (MU and OM), and group B including two reviewers (AH and MO). During the screening process, the blind was turned on in Rayyan to minimize bias during the selection process. First, the records were screened based on title and abstract. In the second step, the blind was removed, and the selection was compared. The final decision was made after discussion. In case of any disagreement, a fifth reviewer (AD) was involved. Next, a full-length screening was performed. Finally, the data were extracted from the included studies in an Excel file regarding participants’ demographics and patient outcomes.

Quality Assessment

Two reviewers independently analyzed each included study and assessed its quality using the Newcastle-Ottawa Scale (NOS) and Joanna Briggs Institute (JBI) risk of bias tools. The NOS checklist has eight questions spread across three categories, which examine how well the study cohort represents the general population, whether confounding factors are controlled, and biases in measuring outcomes. On the other hand, the JBI tool assesses study quality through eight questions for cross-sectional studies.

Results

Included Studies

**Figure 1:**
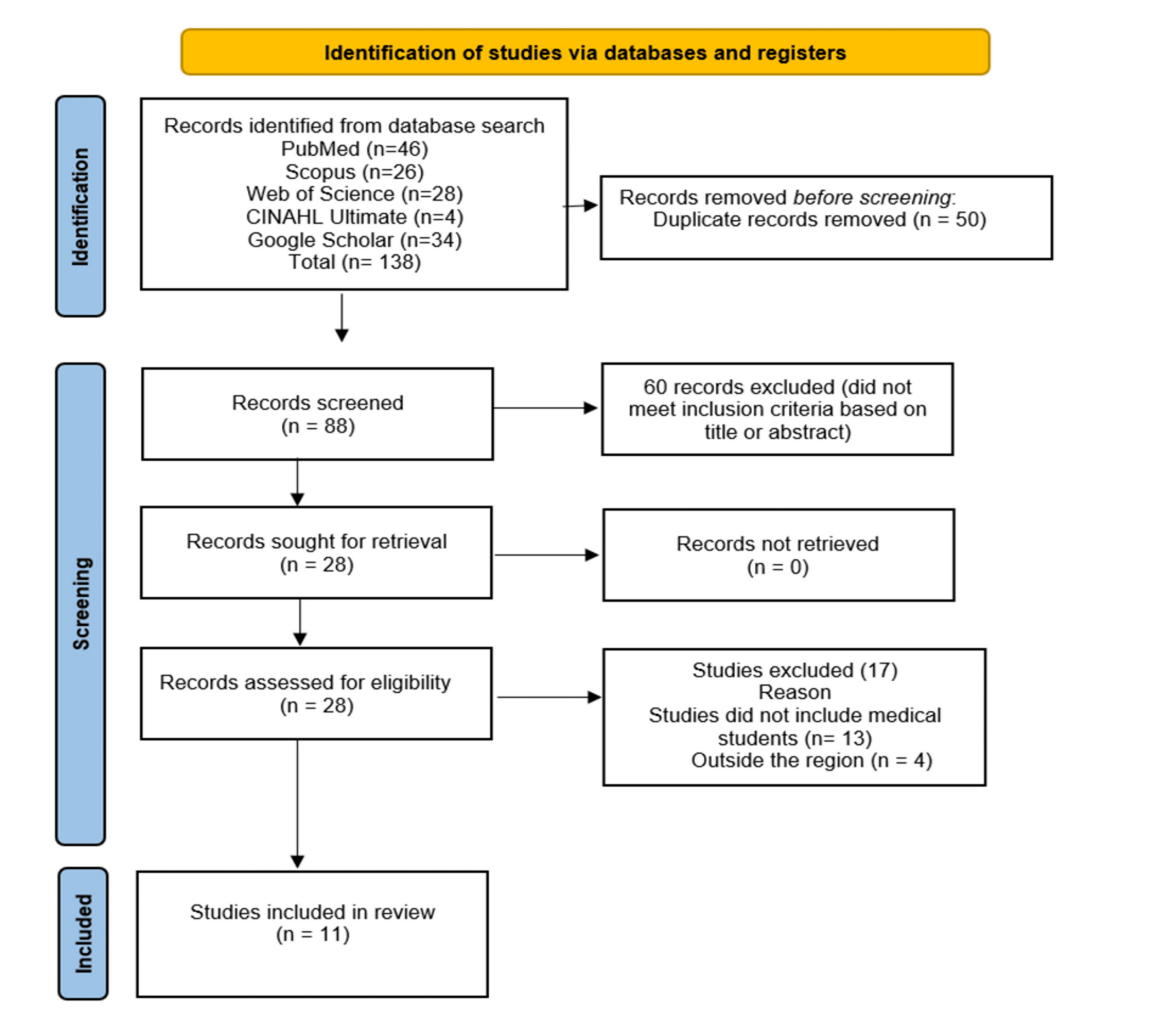
Preferred Reporting Items for Systematic Reviews and Meta-Analyses (PRISMA) flow diagram.

The literature search provided 138 studies from PubMed (n = 46), Web of Science (n = 28), Scopus (n = 26), CINAHL Ultimate (n = 4), and Google Scholar (n = 34). A total of 50 duplicates were removed before the screening process. During screening, 60 records were removed based on title and abstract screening. Based on the predefined inclusion criteria, only 11 studies were included in the systematic review. Figure 1 shows the PRISMA flow diagram of this systematic review.

Study Characteristics and Level of Evidence

A total of 11 studies with 3,063 participants were included in the systematic review. The majority of these studies (9 out of 11) utilized a cross-sectional design, while one study adopted a case-control approach and another employed a prospective study design. Most participants were in their early 20s, with an average age of around 21 years. Females were more common in the included studies than males. Acne was highly prevalent among the participants, with reported rates ranging from 8.3% to 97.9%, with an average of 64.3%. However, in the study by Bouraqqdi et al. (2025), only individuals with acne were included; thus, it was excluded from the pooled prevalence. A consistent pattern emerged concerning stress: nine studies found a statistically significant association between elevated stress levels and increased acne prevalence or severity, generally reporting p-values below 0.05. The remaining two studies did not demonstrate a significant association; however, they still observed that acne severity tended to increase with higher stress levels [1,16]. Various tools were employed to measure acne severity and stress, including the Global Acne Grading System, Cardiff Acne Disability Index, Dermatology Life Quality Index, and the Perceived Stress Scale (Table 1).

**Table 1:** Characteristics of the included studies. GAGS: Global Acne Grading System; AFHC: Adolescent Food Habit Checklist; DLQI: Dermatology Life Quality Index; N/A: not available

Author and year	Country	Study design	Number of participants	Gender	Age range	Prevalence of acne	Effect of stress on acne prevalence	Conclusion
Bouraqqdi et al. (2025) [10]	Morocco	Prospective study	80	51 females, 29 males	Mean: 21.5 years	100%	50% of the students reported stress-related acne flare-ups. During stress, all students had acne. A significant association was found between increased stress and acne severity (p < 001)	These findings support the significant stress-acne correlation
Dabash et al. (2024) [1]	Plestine	Cross-sectional study	350	N/A	Mean: 21.3 ± 1.9 years	80.9%	49.7% of students reported stress, which was associated with the Cardiff Acne Disability Index, with a p-value of 0.005	Stressed students had a high acne severity score, but the difference was not significant
Alayank et al. (2023) [16]	Turkey	Cross-sectional	105	55 females, 50 males	Mean: 21.44 ± 1.69 years	74.28%	31.42% of the medical students were moderately stressed, but there was no statistically significant correlation between PSS and acne severity (p > 0.05)	Although not statistically significant, the PSS was found to be high as the severity of acne increases
Basfar et al. (2023) [8]	Saudi Arabia	Cross-sectional study	585	313 females, 272 males	Mean: 21.1 ± 1.81 years	82.2%	11.8%, 78.5%, and 9.7% of participants had high, moderate, and low stress levels, respectively. Participants with severe stress levels had significantly higher mean GAGS scores; on the other hand, they had significantly lower AFHC scores (p < 0.05)	A positive correlation was found between stress and the severity of acne
Alraddadi et al. (2022) [17]	Saudi Arabia	Cross-sectional survey study	301	179 females, 122 males	Mean: 21.7 years	71.1%	20.7 5.7% of students had moderate-to-severe stress. Moderate-to-severe stress is significantly associated with the development of acne (p < 0.001)	Stress is a significant risk factor for acne prevalence
Aziz et al. (2022) [18]	Saudi Arabia	Cross-sectional study	168	149 females, 19 males	22–25 years	NA	31.54%, 58.34%, and 10.12% of students had low, moderate, and severe stress, respectively. Academic stress had a significant positive association with acne (p < 0.01)	Academic stress is a significant factor in acne in medical students
Khaleel et al. (2022) [19]	Iraq	Case control study	300	83 females, 67 males	18–23 years	50%	Participants with stress had a 2.7 times higher risk of developing acne	The participants’ psychological status (stress) significantly affected acne formation
Alkhawaja (2019) [20]	Oman	Cross-sectional survey	75	50 females, 35 males	26–30 years	8.3%	73% of the students were found to have stress due to acne	Stress associated with acne problems were significantly associated with DLQI scores (p < 0.05)
Ibrahim et al. (2019) [21]	Saudi Arabia	Cross-sectional study	400	N/A	Mean: 21.3 ± 1.4 years	58.8%	72.2% of participants were found to have stress. Stress has a significant association with acne (p < 0.001)	Stress was the most significant predictor of acne. Students suffering from stress were 6.5 times more likely to have acne than others
Allayali et al. (2017) [22]	Saudi Arabia	Cross-sectional study	555	280 females, 275 males	18–24 years	55%	72.1% of students were reported to have psychological stress. The majority of the participants (86.1%) believed that psychological stress is a provoking factor for acne	Acne also has a psychological impact on individuals
Zari and Alrahmani et al. (2017) [12]	Saudi Arabia	Cross-sectional study	144	144 females	22–24 years	97.9%	Stress was strongly correlated with the severity of the acne (p < 0.01)	An increase in stress increases the severity of acne

Quality Assessment of the Included Studies

Table 2 shows the risk of bias measured with the NOS tool for prospective studies and case-control studies. In both studies, the risk of bias was low [10,19].

**Table 2:** Risk of bias measured with Newcastle-Ottawa Scale (NOS).

Study	Selection				Comparability		Outcome			
	Representativeness of the exposed cohort	Selection of the non-exposed cohort	Ascertainment of exposure	Demonstration that the outcome of interest was not present at the start of the study	Controls for the most important risk factors	Controls for other risk factors	Assessment of the outcome	Was the follow-up long enough for outcomes to occur?	Adequacy of follow-up of cohorts	Total quality score
Bouraqqdi et al. (2025) [10]	1	0	1	0	1	1	1	1	1	7
Khaleel et al. (2022) [19]	1	1	1	1	0	0	1	1	1	7

Table 3 shows the risk of bias assessed using the JBI tool for cross-sectional studies. In all studies, the inclusion criteria were clearly defined. However, in two studies, details of the participants were not clearly reported [16,20]. The greatest risk of bias was observed in the cofounding factors domain and strategies to deal with cofounding factors.

**Table 3:** Risk of bias measured with the Joanna Briggs Institute (JBI) tool.

	Dabash et al. (2024) [1]	Alayank et al. (2023) [16]	Basfar et al. (2023) [8]	Alraddadi et al. (2022) [17]	Aziz et al. (2022) [18]	Alkhawaja (2019) [20]	Ibrahim et al. (2019) [21]	Allayali et al. (2017) [22]	Zari and Alrahmani et al. (2017) [12]
Were the criteria for inclusion in the sample clearly defined?	Yes	No	Yes	Yes	Yes	Yes	Yes	Yes	Yes
Were the study subjects and the setting described in detail?	Yes	No	Yes	Yes	Yes	No	Yes	Yes	Yes
Was the exposure measured in a valid and reliable way?	Yes	Yes	Yes	Yes	Yes	Yes	Yes	Yes	Yes
Were objective, standard criteria used for measurement of the condition?	Yes	Yes	Yes	Yes	Yes	Yes	Yes	Yes	Yes
Were confounding factors identified?	No	No	No	No	Yes	No	Yes	No	Yes
Were strategies to deal with confounding factors stated?	Unclear	Unclear	Unclear	Unclear	Yes	No	Yes	No	Yes
Were the outcomes measured in a valid and reliable way?	Yes	Yes	Yes	Yes	Yes	Yes	Yes	Yes	Yes
Was an appropriate statistical analysis used?	Yes	Yes	Yes	Yes	Yes	Yes	Yes	Yes	Yes

Discussion

The findings of this systematic review, based on the analysis of 3,063 medical students from 11 studies, showed a high prevalence of acne 64.3% and a consistent association with stress. The reviewed studies demonstrated a statistically significant correlation between stress levels and acne severity. To our knowledge, this is the first systematic review that has investigated the association between acne and stress among medical students in the Middle East. Prior reviews, such as systematic reviews of acne guidelines and meta-analyses, have explored the association between stress and acne globally; none have specifically addressed this demographic region [23,24].

The findings of the present systematic review are supported by the systematic review and meta-analysis by Salari et al., which analyzed the prevalence of stress and other psychological conditions in people with skin disorders, including acne. Their findings showed that 75.7% of the acne participants were suffering from stress. This study included 113 studies that provide a robust dataset for meta-analysis, enhancing the reliability of the pooled prevalence estimates, but was limited due to the high heterogeneity and cross-sectional data predominance [24]. Similarly, our findings also align with a literature review that specifically included medical students [4].

A recent literature review, including 13 cross-sectional studies, found that acne prevalence among medical students ranged from 34.38% to 97.9% [4]. The findings of this study were also aligned with the present systematic review. This study found that stress is a risk factor for developing acne. This study specifically targeted medical students, as in the present study. Medical students experience high stress. The included studies cover a diverse range of countries, enhancing the generalizability of findings across different cultures and settings. The study reported the prevalence and additionally synthesized data on psychological and social impacts. In addition to stress, gender and lifestyle were also significant factors. However, this study was limited by the variability of the assessment methods, as there was an inconsistency in how acne was diagnosed, which may affect the comparability and pooled prevalence estimates. All the included studies were cross-sectional, which restricts the ability to infer causality between risk and acne. Not all the included studies reported psychosocial comorbidities [4].

The present systematic review also found a bidirectional relationship between acne and stress. It has been previously reported in the literature that acne contributes to psychological distress, social withdrawal, and reduced self-esteem, which, in turn, may worsen acne through the neuroendocrine pathway [4,25]. Stress is a menace to a person’s welfare, and young adults often experience stress due to numerous factors, such as financial insecurity, lack of job security, lack of time management, and academic pressures [26]. An irresistible requirement to perform well in studies leads to academic stress that can impair the student’s morale, cognition, and psychological state [18].

Stress occurs due to a stimulus that induces reactions in the brain and is one of the acne triggers. There is strong evidence that supports the correlation between stress and acne centered on the HPA axis [27]. The HPA axis is a neuroendocrine system that regulates the immune responses and the nervous system’s reaction to stressors [28]. It affects the function of the sebaceous glands by a precise mechanism. The sebaceous glands in the skin have corticotropin-releasing hormone receptors. Stress increases the levels of corticotropin-releasing hormone, which stimulates the formation of sebum. Sebum clogs the pilosebaceous gland ducts [27]. In addition to expressing the corticotropin-releasing hormone receptor, sebaceous follicular glands can also contain neuropeptide receptors such as vasoactive intestinal polypeptide, calcitonin gene-related peptide, neuropeptide Y, β-endorphin, and α-melanosyte stimulating hormone. The binding of the neuropeptides to their receptors causes an increase in the production of proinflammatory cytokines such as interleukin (IL)-1, IL-6, tumor necrosis factor-α, and peroxisome proliferator-activated receptors-γ. The increased production of these cytokines results in proliferation, differentiation, and lipogenesis of the pilosebaceous glands, ultimately causing acne on the skin [27,29].

Managing the triggers of acne is essential to controlling its development. It has been reported that psychological interventions, mainly the biofeedback-assisted relaxation imagery, have the potential to improve skin conditions by directly targeting the stress-related physiological responses. Additionally, teaching individuals to control stress-induced physiological arousal, biofeedback therapy can decrease the activation of the HPA axis and lower cortisol levels, leading to reduced inflammation and acne severity [30].

Strengths and limitations

There are several strengths of this systematic review. First, this systematic review adopted a systematic search in various databases to identify relevant studies, which highlights a comprehensive search strategy used in the review process. Second, it is the first systematic review to investigate acne and stress among medical students in the Middle East. However, there are some limitations as well. First, a meta-analysis was not performed. Second, the majority of the studies were cross-sectional, with only one study having a prospective cohort design. For this reason, reliance on self-reported stress and acne outcomes could be prone to reporting bias. Furthermore, the majority of the studies had a small sample size. Language restrictions and the exclusion of non-English-language articles may have omitted relevant regional research.

Implications for research and practice

Future research should prioritize longitudinal and interventional studies to clarify the directionality of the stress-acne relationship and establish causality. Standardization of assessment tools for both stress and acne severity will enhance comparability across studies and enable meta-analytic pooling. Investigations into biological mechanisms, such as stress-induced hormonal changes affecting sebaceous gland activity, could inform targeted therapies. In clinical practice, routine screening for stress levels among medical students presenting with acne may help identify those who would benefit from early psychosocial interventions.

## Conclusions

The collective evidence from Middle Eastern medical student populations indicates a clear association between elevated stress levels and increased prevalence and severity of acne vulgaris. Although most included studies were cross-sectional, the consistency of findings across diverse settings supports the importance of addressing psychological stress as part of acne management in young adults. Implementing standardized research methodologies and designing longitudinal studies will strengthen causal understanding, while embedding stress-reduction interventions within medical training may offer dual benefits for skin health and academic performance.

## Appendices

**Table 4:** Search strategy.

Key Variable	Sub terms	Search options	PubMed	Web of Science	Scopus	CINAHL Ultimate
1. Stress	#1 Stress	TI/AB	1,127,806	2,259,515	2,859,875	190,155
	#2 Psychological stress	TI/AB	12,121	63,417	86,021	7,196
	#3 Mental stress	TI/AB	4,412	66,675	85,180	5,635
	#4 Emotional stress	TI/AB	5,593	41,008	55,059	3,852
	#5 Psychosocial stress	TI/AB	5,478	21,290	25,860	2,543
	#6 Anxiety	TI/AB	310,050	362,672	445,136	117,580
	#7 Academic stress	TI/AB	821	16,988	25,649	956
((Stress[Title/Abstract]) OR (Psychological stress[Title/Abstract])) OR (Mental stress[Title/Abstract])) OR (Emotional stress[Title/Abstract])) OR (Psychosocial stress[Title/Abstract])) OR (Anxiety[Title/Abstract])) OR (Academic stress[Title/Abstract])			1m372,157	2,552,439	3,218,975	285,444
2. Acne	#1 Acne	TI/AB	21,649	25,250	33,220	4,972
	#2 Acne vulgaris	TI/AB	6,302	5,915	8,188	1,052
	#3 Facial acne	TI/AB	772	1,872	2,487	219
((Acne[Title/Abstract]) OR (Acne vulgaris[Title/Abstract])) OR (Facial acne[Title/Abstract])			21,649	25,250	33,220	4,972
3. Middle East	#1 Middle East	Mesh	178,743			
	#2 Middle East	TI/AB	19,212	62,568	97,136	4,379
	#3 Iran	TI/AB	62,066	120,489	179,964	23,226
	#4 Saudi Arabia	TI/AB	33,397	53,587	69,733	6,793
	#5 United Arab Emirates	TI/AB	4,654	9,993	16,271	1,331
	#6 Israel	TI/AB	30,746	75,297	93,760	8,945
	#7 Lebanon	TI/AB	7,317	14,241	19,577	2,289
	#8 Jordan	TI/AB	10,425	35,411	50,732	3,814
	#9 Bahrain	TI/AB	1,600	3,339	7,203	451
	#10 Oman	TI/AB	4,712	12,435	19,673	1,227
	#11 Qatar	TI/AB	4,123	7,793	13,482	1,322
	#12 Yemen	TI/AB	2,640	5,856	9,082	676
	#13 Syria	TI/AB	3,608	12,980	19,848	1,004
	#14 Egypt	TI/AB	21,321	57,613	84,066	3,480
	#15 Iraq	TI/AB	10,093	27,204	56,068	4,245
	#16 Kuwait	TI/AB	4,893	10,726	17,887	1,261
	#17 Palestine	TI/AB	2,719	13,027	17,402	1,038
((“Middle East”[Mesh]) OR (Middle East[Title/Abstract])) OR (Iran[Title/Abstract])) OR (Saudi Arabia[Title/Abstract])) OR (United Arab Emirates[Title/Abstract])) OR (Israel[Title/Abstract])) OR (Lebanon[Title/Abstract])) OR (Jordan[Title/Abstract])) OR (Bahrain[Title/Abstract])) OR (Oman [Title/Abstract])) OR (Qatar[Title/Abstract])) OR (Yemen[Title/Abstract])) OR (Syria[Title/Abstract])) OR (Egypt [Title/Abstract])) OR (Iraq[Title/Abstract])) OR (Kuwait[Title/Abstract])) OR (Palestine[Title/Abstract])			295,090	478,314	688,571	61,516
Final combined results across all key variables			46	28	26	4

## References

[1] Dabash D, Salahat H, Awawdeh S, Hamadani F, Khraim H, Koni AA, Zyoud SH (2024). Prevalence of acne and its impact on quality of life and practices regarding self-treatment among medical students. Sci Rep.

[2] Sutaria AH, Masood S, Saleh HM, Schlessinger J (2023). Acne Vulgaris. https://www.ncbi.nlm.nih.gov/books/NBK459173/.

[3] Vasam M, Korutla S, Bohara RA (2023). Acne vulgaris: a review of the pathophysiology, treatment, and recent nanotechnology based advances. Biochem Biophys Rep.

[4] Sachdeva M, Tan J, Lim J, Kim M, Nadeem I, Bismil R (2021). The prevalence, risk factors, and psychosocial impacts of acne vulgaris in medical students: a literature review. Int J Dermatol.

[5] Alrabiah Z, Arafah A, Rehman MU (2022). Prevalence and self-medication for acne among students of health-related science colleges at King Saud University in Riyadh region Saudi Arabia. Medicina (Kaunas).

[6] Al-Kubaisy W, Abdullah NN, Kahn SM, Zia M (2014). Sociodemographic characteristics of acne among university students in Damascus, Syria. Epidemiol Res Int.

[7] Lim TH, Badaruddin NS, Foo SY, Bujang MA, Muniandy P (2022). Prevalence and psychosocial impact of acne vulgaris among high school and university students in Sarawak, Malaysia. Med J Malaysia.

[8] Basfar AS, Jawhari AM, Alotaibi MN, Alzahrani ES, Aseeri IA, Atalla AA (2023). Severity of acne, stress, and food habits of medical students at Taif University, Saudi Arabia. J Family Community Med.

[9] Almojali AI, Almalki SA, Alothman AS, Masuadi EM, Alaqeel MK (2017). The prevalence and association of stress with sleep quality among medical students. J Epidemiol Glob Health.

[10] Bouraqqadi O, Soughi M, Maiouak M, Douhi Z, Elloudi S, BayBay H, Mernissi FZ (2025). The impact of academic stress on acne: an observational cohort study among medical students in Morocco. JAAD Int.

[11] Suh DH, Kim BY, Min SU (2011). A multicenter epidemiological study of acne vulgaris in Korea. Int J Dermatol.

[12] Zari S, Alrahmani D (2017). The association between stress and acne among female medical students in Jeddah, Saudi Arabia. Clin Cosmet Investig Dermatol.

[13] Alanazi AZJ (2020). Acne vulgaris in Saudi Arabia: a systematic review. J Dermatol Dermatol Surg.

[14] Moher D, Shamseer L, Clarke M (2015). Preferred reporting items for systematic review and meta-analysis protocols (PRISMA-P) 2015 statement. Syst Rev.

[15] Ouzzani M, Hammady H, Fedorowicz Z, Elmagarmid A (2016). Rayyan-a web and mobile app for systematic reviews. Syst Rev.

[16] Alyanak A, Çavdar Ü, Emir B (2023). Investigation of the effect of stress and other factors on acne in medical students. Int J Sci Res Arch.

[17] Alraddadi M, Alanazi AMM, Al-Ghamdi FSA (2022). Acne vulgaris and its association with stress, sleep deprivation, and dietary intake among medical Students in Tabuk University, KSA. Bull Natl Inst Health Sci.

[18] Aziz F, Khan MF (2022). Association of academic stress, acne Symptoms and other physical symptoms in medical students of King Khalid University. Int J Environ Res Public Health.

[19] Khaleel FF (2022). Risk factors of acne vulgaris among Mosul University students from Iraq. Iraqi J Med Sci.

[20] Alkhawaja A (2019). The quality of life and psychological impact of acne vulgaris among university students in Nizwa-Oman. Int J Novel Res Healthcare Nurs.

[21] Ibrahim NK, Nagadi SA, Idrees HJ, Alghanemi LG, Essa RI, Gari WS (2019). Acne vulgaris: prevalence, predictors, and factors influencing quality of life of female medical students at King Abdulaziz University, Jeddah. J Dermatol Dermatol Surg.

[22] Allayali AZ, Asseri BN, AlNodali NI, Alhunaki RNM, Algoblan SG (2017). Assessment of prevalence, knowledge, attitude, and psychosocial impact of acne vulgaris among medical students in Saudi Arabia. J Clin Exp Dermatol Res.

[23] Corcoran L, Muller I, Layton AM (2023). Systematic review of clinical practice guidelines for acne vulgaris published between January 2017 and July 2021. Skin Health Dis.

[24] Salari N, Heidarian P, Hosseinian-Far A, Babajani F, Mohammadi M (2024). Global prevalence of anxiety, depression, and stress among patients with skin diseases: a systematic review and meta-analysis. J Prev (2022).

[25] Sharif S, Tahir S, Malik AF (2024). Prevalence of acne and its association with stress in female medical students. Pak J Med Dent.

[26] Kohli N, Dua K (2022). Stress among youth: causes and its management in recent times. Int J Sci Res.

[27] Jusuf NK, Putra IB, Sutrisno AR (2021). Correlation between stress scale and serum dubstance P level in acne vulgaris. Int J Gen Med.

[28] Sheng JA, Bales NJ, Myers SA, Bautista AI, Roueinfar M, Hale TM, Handa RJ (2020). The hypothalamic-pituitary-adrenal axis: development, programming actions of hormones, and maternal-fetal interactions. Front Behav Neurosci.

[29] Yosipovitch G, Tang M, Dawn AG, Chen M, Goh CL, Huak Y, Seng LF (2007). Study of psychological stress, sebum production and acne vulgaris in adolescents. Acta Derm Venereol.

[30] Seung J (2024). Balancing acne: the review of psychological and physical therapies in stressful rnvironments. Natl High Sch J Sci.

